# Psychometric validation of the EORTC QLQ-OES18 in patients with advanced or metastatic esophageal squamous cell carcinoma

**DOI:** 10.1186/s41687-025-00891-4

**Published:** 2025-05-21

**Authors:** Lauren Podger, Daniel Serrano, Liyun Li, Lin Zhan, Boxiong Tang, Gisoo Barnes

**Affiliations:** 1 Open Health Group, London, UK; 2 The Psychometrics Team, Sheridan, WY USA; 3 BeOne Medicines Ltd, San Carlos, CA USA

**Keywords:** Esophageal squamous cell cancer, Patient-reported outcomes, Psychometrics, Validation, Health-related quality of life, EORTC QLQ-OES18

## Abstract

**Background:**

The EORTC QLQ-OES18 has previously demonstrated clinical validity; however, there are limited published psychometric data for patients with advanced esophageal squamous cell carcinoma (ESCC). We evaluated the measurement properties of the QLQ-OES18 in a clinical trial population of patients with advanced or metastatic ESCC.

**Methodology:**

Analyses used data from RATIONALE 302 (NCT03430843), a randomized phase 3 study of tislelizumab versus investigator-chosen chemotherapy as second-line treatment for patients with advanced or metastatic ESCC. Psychometric validation of the QLQ-OES18 included tests of reliability, construct validity, ability to detect change, and estimation of anchor-based meaningful within-patient change (MWPC) thresholds—the latter two being exploratory given that the trial was not powered to detect efficacy in patient-reported outcome endpoints.

**Results:**

In total, 512 patients were randomized to either tislelizumab or chemotherapy; the average age was 61.5 years, and 84.4% were male. Three of the 4 QLQ-OES18 multi-item scales (dysphagia, eating, and pain) and the index scale met the prespecified criterion for acceptable internal consistency as well as acceptable test-retest reliability. Associations between baseline QLQ-OES18 scores and convergent/discriminant validators were generally as expected (i.e., the QLQ-OES18 pain score had a strong positive correlation with the QLQ-C30 pain score). For known-groups validity, 88.6% of analyses demonstrated the hypothesized direction of effect, suggesting that the expected differences in baseline QLQ-OES18 scores between prespecified groups were observed. Ability to detect change analyses indicated that several QLQ-OES18 domain scores demonstrated sensitivity in detecting possible treatment effects, although many patients reported minimal symptoms at baseline, which limited the ability to detect significant improvement.

**Conclusion:**

Overall, a collection of psychometric evidence indicated that the EORTC QLQ-OES18 reliably and validly measured symptom severity in the RATIONALE 302 population. Specifically, the dysphagia domain consistently demonstrated robust psychometric properties. Limitations in data reduced the interpretability of MWPC thresholds and are discussed in detail.

**Supplementary Information:**

The online version contains supplementary material available at 10.1186/s41687-025-00891-4.

## Background

Esophageal squamous cell carcinoma (ESCC), the most common histological subtype of esophageal cancer, frequently results in a high patient burden at diagnosis, as well as associated reductions in health-related quality of life (HRQoL) due to esophageal obstruction throughout the disease course, including during treatment [1–3]. HRQoL and other self-reported symptomatic and health status measures are collected via patient-reported outcome measures (PROMs) [4]. Among patients with potentially curable and advanced esophageal cancer, such PROMs have been shown to be predictive of overall survival [5, 6]. The association between overall survival and PROMs emphasizes the importance of capturing these data; however, less is known about the relationship between PROMs and survival in treatment-refractory populations. Given the shift toward novel molecules as second-line standard of care for ESCC [4, 7–9], the potential for PROMs to capture changes in key symptoms in treatment-refractory populations has arisen. With this potential comes the need to understand the measurement properties of such PROMs in these populations.

Several different PROMs have been employed to measure HRQoL in studies of ESCC, with the European Organisation for Research and Treatment of Cancer (EORTC) Quality of Life Questionnaire – Oesophageal Cancer 18-question module (QLQ-OES18) frequently used. Although the QLQ-OES18 has previously demonstrated clinical validity [10], there are limited published studies that have examined the measurement properties of this instrument in patients with advanced or metastatic ESCC. Furthermore, when evaluating the meaningfulness of changes in EORTC questionnaire scores over time, a 10-point threshold has historically been cited [11]. However, this threshold was derived from a sample of several cancer types (e.g., lung and breast cancer) and specifically for select EORTC Quality of Life Questionnaire – Core 30 (QLQ-C30) domain scores [12]. The approach used to estimate the historical 10-point threshold is inconsistent with current guidance from the US Food and Drug Administration (FDA); the approach has not been confirmed to generalize across the various EORTC disease-specific measures, and anchors employed in establishing these thresholds are not acceptable to the FDA based on current recommendations [13, 14].

The objective of the current analysis was to evaluate the measurement properties of the EORTC QLQ-OES18 instrument in the RATIONALE 302 clinical trial population, while recognizing the limited published psychometric evidence and the need for estimation of anchor-based within-patient thresholds.

## Methods

RATIONALE 302 (NCT03430843), a global, open-label, randomized, phase 3 study, investigated the efficacy and safety of tislelizumab, an anti–programmed cell death 1 protein monoclonal antibody, versus that of investigator-chosen chemotherapy as second-line treatment for patients with advanced or metastatic ESCC that progressed after first-line systemic therapy. HRQoL and patient-reported ESCC symptoms were secondary endpoints in RATIONALE 302 and were assessed using PROMs. Detailed methodology for RATIONALE 302 has previously been published [15]. As with most oncology trials, RATIONALE 302 was not powered to detect efficacy on PRO endpoints and none was observed. Relevant details for the psychometric analyses are summarized below.

### Study design and population

Patients were randomized (1:1) to receive tislelizumab (220 mg every 3 weeks) or a single-agent chemotherapy (paclitaxel, docetaxel, or irinotecan), as selected by the investigator; all treatment was received on an open-label basis. To be eligible, patients were required to be aged ≥ 18 years with histologically confirmed ESCC and advanced or metastatic disease that progressed during or after first-line systemic treatment. Patients who had tumor progression during or within 6 months after definitive chemoradiotherapy or neoadjuvant or adjuvant therapy were also eligible. Requirements included an Eastern Cooperative Oncology Group (ECOG) performance status of 0 or 1; ≥1 measurable/evaluable lesion by Response Evaluation Criteria in Solid Tumors version 1.1; and adequate hematologic, hepatic, renal, and coagulation function. Exclusion criteria included receipt of prior therapies targeting programmed cell death 1 protein, active brain or leptomeningeal metastasis, active autoimmune disease, or other prior malignancies active within 2 years before randomization.

### Patient-reported outcome measures

The EORTC QLQ-OES18 [10] and QLQ-C30 [16] instruments were included as part of the RATIONALE 302 schedule of assessments. The EORTC QLQ-OES18 is a self-report 18-item questionnaire designed to assess HRQoL in patients with esophageal cancer [10]. This PROM consists of 4 multi-item symptom scales: dysphagia, eating, reflux, and pain. It also consists of 6 symptom single items: swallowing saliva, choking when swallowing, dry mouth, trouble with taste, trouble with coughing, and trouble with talking. In addition, an index scale is calculated as a composite of all 18 item scores. These items are scored using a verbal-descriptive scale rated from 1 to 4 (“not at all,” “a little,” “quite a bit,” and “very much”) during the past week. Transformed scores from each scale and single item range from 0 to 100; higher symptom scores indicate worse symptoms or reduced HRQoL. Although the dysphagia scale is scored as a functional scale, with higher scores indicating better function/health, the scoring of the dysphagia scale was reversed to maintain consistency with the rest of the symptom domains. The QLQ-OES18 was the focus of these validation analyses.

The 30-item EORTC QLQ-C30 is a well-established generic measure for evaluating the quality of life in patients with cancer across physical, emotional, and social health issues [16, 17]. This PROM includes 15 domains: global health status/quality of life (GHS/QoL), physical functioning, role functioning, emotional functioning, cognitive functioning, and social functioning. The following symptoms are also included: fatigue, nausea and vomiting, pain, dyspnea, insomnia, appetite loss, constipation, diarrhea, and financial difficulties. An index scale is also calculated as a composite of all 15 domain scores. The QLQ-C30 employs a past-week recall period; the 2 GHS/QoL items are scored using a numeric rating scale from 1 to 7 (“very poor” to “excellent”). Conversely, the remaining items are scored using a verbal-descriptive scale from 1 to 4 (“not at all,” “a little,” “quite a bit,” and “very much”). Transformed scores from each domain range from 0 to 100. Higher scores on the GHS/QoL and functional scales represent a higher global quality of life or functioning level. In contrast, higher scores for the symptom domains and index scale represent a more severe symptom/problem.

The QLQ-C30, geographic region (Asia versus US/EU), and ECOG performance were used as anchors and/or validators of the QLQ-OES18, described in detail within each analysis section.

### Psychometric analyses

All psychometric analyses were conducted in a cohort of randomized patients with complete QLQ-OES18 and QLQ-C30 data at baseline. For the sake of brevity, ability to detect change and estimation of anchor-based meaningful within-patient change (MWPC) thresholds are referred to globally using the term “responsiveness.” Note, however, that this term should not be interpreted to mean that these analyses were designed to yield responder definitions. Instead, “responsiveness” analyses were designed only to estimate and evaluate within-arm meaningful change thresholds. Key time points included the baseline visit (Cycle 1 Day 1; start of treatment), first follow-up visit at week 3 (Cycle 2 Day 1) for test-retest reliability estimation, and follow-up visit at week 9 (Cycle 4 Day 1) for estimation of responsiveness.

Missing data for the QLQ-OES18 and QLQ-C30 items were handled according to the developer’s manuals [10, 18]. All analyses were performed using SAS (version 9.4) and R statistical software (version 4.0.3). Table 1 provides a summary of the psychometric analyses that were conducted, which are described in detail below.

**Table 1:** Summary of primary psychometric analyses of QLQ-OES18

Property	Analysis Period	Definition	Test	Success Criterion
Item response distributions	Baseline	n (%)	No test	---
Inter-item correlations	Baseline	Polychoric correlation	No test, point estimate reported	|r| ≥ 0.40
Internal consistency	Baseline	Cronbach alpha	No test, point estimate reported	α ≥ 0.70
Test-retest reliability	Baseline to week 3	ICC(A,1)	No test, point estimate reported	ICC(A,1) ≥ 0.70
Concurrent validity	Baseline	Spearman correlation	No test, point estimate reported	|r| ≥ 0.40
Known-groups validity	Baseline	Mean difference; 95% CI, p-value, and $\:{\omega\:}^{2}$ effect size	ANOVA	*P* < 0.05; effect size > 5%
Ability to detect change	Baseline to week 9	Mean difference; 95% CI, p-value, and $\:{\omega\:}^{2}$ effect size	MMRM	*P* < 0.05; effect size > 5%
Meaningful within-patient change	Baseline to week 9	Mean/median change from baseline and eCDFs/ePDFs plotted	No test, point estimate reported	---

Note. Analyses were conducted using transformed scores on both the QLQ-OES18 and QLQ-C30

Abbreviations. ANOVA: analysis of variance; CI: confidence interval; eCDF: empirical cumulative distribution function; ePDF: empirical probability density function; ICC: intraclass correlation coefficient; MMRM: mixed model for repeated measures; QLQ-C30: Quality of Life Questionnaire – Core 30; QLQ-OES18: Quality of Life Questionnaire – Oesophageal Cancer 18-question module

Descriptive assessments for the QLQ-OES18 items were conducted on the total sample (i.e., pooled treatment arms) at baseline. Sparseness of response distributions (categories endorsed by < 10% of the sample) as well as floor and ceiling effects (e.g., 25% response in lowest or highest category) were evaluated. Substantial ceiling and/or floor effects may indicate a need to reduce the number of response categories [19]. Additionally, inter-item correlations were estimated between the QLQ-OES18 items; an inter-item correlation of ≥ 0.40 indicated acceptable shared variance.

### Reliability

Internal consistency was assessed for each of the QLQ-OES18 multi-item domains at baseline using Cronbach alpha [20]. Item-level internal consistency was characterized using the item-total correlations and item-level Cronbach alpha. Internal consistency estimates of ≥ 0.70 were considered acceptable [21].

Test-retest reliability was calculated in a subset of patients with stable disease, as measured by an external criterion. For this analysis, patients whose responses on the QLQ-C30 GHS/QoL scale reflected no change in status between baseline and the week 3 follow-up were considered to be stable. Test-retest reliability was assessed for each of the QLQ-OES18 domain scores. Estimates were based on absolute-agreement, 2-way mixed-effects intraclass correlation coefficients (ICCs [A,1]); and estimates of ≥ 0.70 indicated satisfactory reliability [22, 23].

### Construct validity

Concurrent validity was estimated by correlating QLQ-OES18 scores with QLQ-C30 scores using Spearman correlations at baseline. Larger correlations (≥ 0.40) reflect convergent validity, whereas small correlations (< 0.40) reflect divergent or discriminant validity [24]. Specific hypotheses for convergent associations between scores included the following: (1) QLQ-OES18 eating and trouble with taste scores would have a moderate to strong positive correlation (0.40–0.80) with the QLQ-C30 appetite loss score, (2) the QLQ-OES18 pain score would have a moderate to strong positive correlation (0.40–0.80) with the QLQ-C30 pain score, and (3) the QLQ-OES18 trouble with coughing score would have a moderate positive correlation (0.40–0.50) with the QLQ-C30 dyspnea score. Based on item content and concepts measured, the expected discriminant validators included the QLQ-C30 constipation, diarrhea, and financial difficulties scores. Any associations beyond these prespecified hypotheses were considered exploratory.

Known-groups validators included geographic region (Asia versus US/EU, as required by the Chinese health authority [NMPA]), ECOG performance status at baseline (0 versus 1) [25], and QLQ-C30 GHS/QoL item scores (ratings of 1–6 versus 7). Note, QLQ-C30 GHS/QoL items 29 and 30 were treated as separate validators. The hypothesized direction of effect predicted that patients in the US/EU would report worse symptoms than patients in Asia, patients with an ECOG performance status of 1 would report worse symptoms than those with an ECOG performance status of 0, and patients selecting “excellent” on the QLQ-C30 GHS/QoL items would report fewer/better symptoms (lower QLQ-OES18 scores) than patients who selected any of the remaining response options.

The difference in QLQ-OES18 scores between each known group was calculated and contrasted using analysis of variance (ANOVA), from which the mean difference between known groups, corresponding 95% confidence interval (CI), p-value, and omega squared (ω^2^) effect size was estimated. The ω^2^ statistic [26] is an effect size estimate related to the commonly employed R^2^, characterizing the proportion of variance in the response variables (e.g., PRO scores) accounted for by the explanatory variables (e.g., geographic region). The ω^2^ statistic is preferred over R^2^ or η2 as it is considered to be the least biased estimator [26, 27]. Acceptable known-groups validity was achieved if a preponderance of the known-effect groups had higher or significantly higher scores, and corresponding effect sizes were > 5%.

### Responsiveness

The responsiveness of the QLQ-OES18 scores was evaluated by ability to detect change and estimation of anchor-based MWPC thresholds [28].

Ability to detect change was assessed by evaluating the relationship between changes in the QLQ-OES18 scores and changes in an external measure that assesses proximal constructs. For this analysis, the QLQ-C30 GHS/QoL scores served as the external measure (i.e., anchor). Each analysis consisted of two anchor-based contrasts testing the difference in QLQ-OES18 change from baseline to week 9 follow-up between each effect group (1-point improvement or 1-point deterioration) against the reference group (maintenance [no change]). The relationship between change in QLQ-OES18 scores and changes in the anchor scores was estimated via mixed models for repeated measures (MMRM). The primary estimate of interest was differences in marginal means for QLQ-OES18 change between anchor groups, adjusting for age, sex, geographic region, and baseline QLQ-OES18 scores. Marginal mean differences in the QLQ-OES18 change scores, along with corresponding standard error (SE), 95% CIs, p-values, and ω^2^ effect sizes, were estimated for each anchor group contrast. Acceptable ability to detect change was prespecified as a significant difference in the marginal means across anchor group contrasts and corresponding effect sizes of > 5%.

In addition, two sensitivity analyses were conducted. First, additional marginal mean contrasts were calculated using a broader anchor change group definition: improvement (≥ 1-point improvement), maintenance (no change), and deterioration (≥ 1-point deterioration). Second, Spearman correlations between QLQ-OES18 change scores (i.e., change from baseline to week 9) and change from baseline in the QLQ-C30 GHS/QoL scores were calculated. Correlations of 0.30 to 0.70 were considered to indicate acceptable ability to detect change [29].

Estimation of MWPC thresholds for the QLQ-OES18 scores was conducted using anchor-based methods, consistent with US FDA draft guidance [14, 30].

Both mean and median change from baseline to the week 9 follow-up were estimated for each of the QLQ-OES18 domains. Estimates were calculated according to anchor group membership defined by change from baseline to week 9 in QLQ-C30 GHS/QoL scores. Anchor groups included 2 or more points of deterioration, 1 point of deterioration, no change, 1 point of improvement, and 2 or more points of improvement.

In the case of transformed scores, like those employed by EORTC measures, the US FDA has asserted that MWPC thresholds corresponding to less than 1 point change on the raw score (2018 PFDD 3 discussion document lines 1108–1111) are not interpretable:

Depending on the proposed score transformation, selected improvement threshold(s) based on transformed scores may reflect less than one category change on the raw score scale, which is not useful for the evaluation and interpretation of clinically meaningful change.

(i.e., 1–4) corresponds to a ± 33.33-point change on the transformed score (i.e., 0-100)^1^ and thus, the minimum interpretable MWPC threshold for the EORTC transformed scores is ± 33.33 according to this criterion recommended by FDA^2^ [30, 31].

**Fig. 1:**
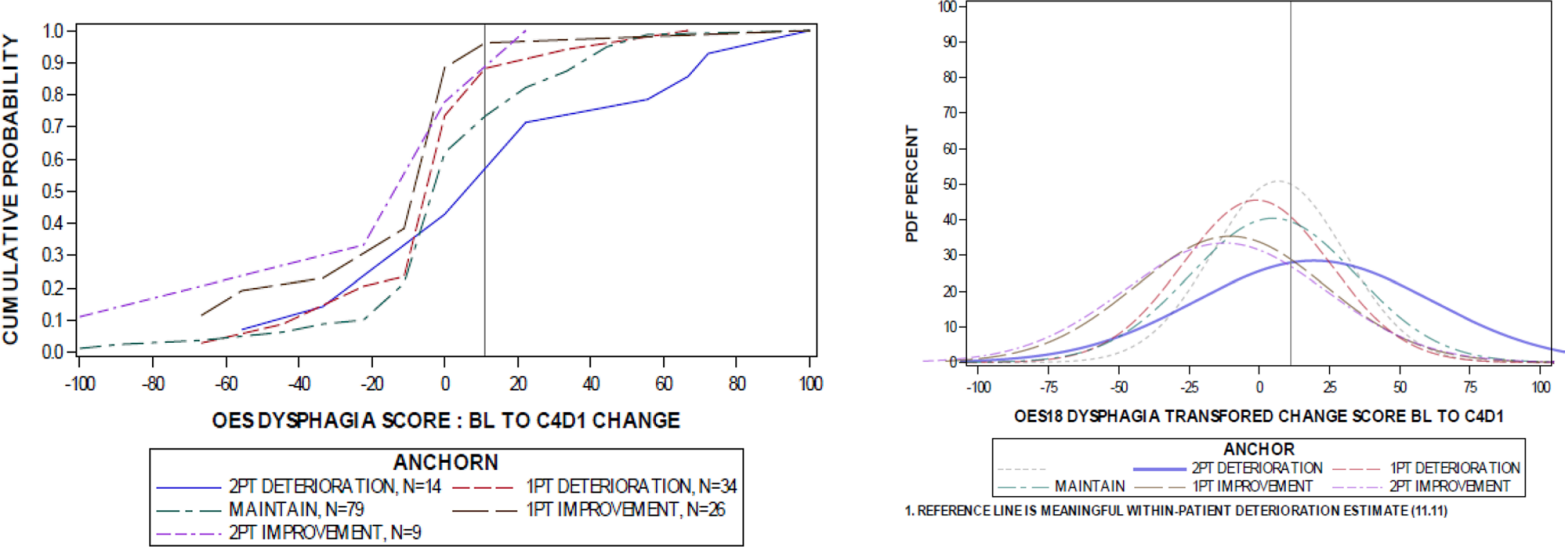
eCDF and ePDF of QLQ-OES18 dysphagia change scores from baseline to week 9 by anchor group. Abbreviations. BL: baseline; C4D1: cycle 4 day 1; eCDF: empirical cumulative distribution function; ePDF: empirical probability density function; PT: point; QLQ-OES18: Quality of Life Questionnaire – Oesophageal Cancer 18-question module.

This criterion is rather stringent and may not be achievable within this trial population, and therefore, the feasibility of such a criterion was examined in this analysis.

Point estimates for median-based thresholds were visually explored by plotting the differences in cumulative proportions achieving the estimated anchor-based MWPC threshold stratified on anchor group membership via empirical cumulative distribution function (eCDF) and empirical probability density function (ePDF) figures.

Distribution-based estimates of 0.5 standard deviation (SD) and standard error of measurement (SEM) were calculated and considered as supplemental evidence of meaningful change.

## Results

Overall, 512 patients were randomized to either tislelizumab (*n* = 256) or chemotherapy (*n* = 256) between January 2018 and March 2020. The intent-to-treat cohort had an average age of 61.5 years, and the majority of patients were male (84.4%), Asian (79.7%), and non-Hispanic (98.4%). Median body mass index was 21.1; 11.5% were current smokers, and 12.7% were current drinkers of alcohol. Approximately one-quarter of the cohort (24.6%) had an ECOG performance status of 0 (fully active), whereas approximately three-quarters (75.1%) had a performance status of 1 (restricted in physically strenuous activity but ambulatory and able to carry out light work). All baseline characteristics were balanced across treatment strata; full demographic and clinical characteristics of the intent-to-treat population at baseline have been published in the primary manuscript [15]. A total of 23 patients were missing PROM data at baseline and were thus excluded from the current psychometric analyses [15].

Response patterns for the QLQ-OES18 at baseline indicated the presence of floor effects for all 18 items (see Supplemental Table 1 in the Electronic supplementary material for complete QLQ-OES18 item response distributions). However, it is important to note that other response categories (e.g., “a little” or “quite a bit”) for these items were reasonably endorsed. The instances of floor effects suggest that this population had lower self-reported symptom severity at baseline. No item endorsements indicated ceiling effects. In terms of inter-item correlations, 54 of 153 (35.3%) met the prespecified criterion for acceptable shared variance. A minority of inter-item correlations were < 0.20. See Supplemental Table 2 in the Electronic supplementary material for the complete inter-item polychoric correlation matrix.

### Reliability

Three of the four QLQ-OES18 multi-item domains demonstrated acceptable internal consistency (α = 0.87, α = 0.77, and α = 0.71 for the dysphagia, eating, and pain domains, respectively); the index score also demonstrated acceptable internal consistency (α = 0.78). The reflux domain did not meet the prespecified criterion, although it was within rounding distance (α = 0.67). The item-total correlations were consistent with the observed trend in score-level alpha estimates. See Supplemental Table 3 in the Electronic supplementary material for the complete listing of item-level Cronbach alpha values.

The test-retest reliability estimates for the QLQ-OES18 are presented in Table 2. The ICC estimates ranged between 0.41 and 0.78 (see Table 2). For the dysphagia, eating, and pain domains, as well as the index score, the ICC estimates exceeded the prespecified criterion of ≥ 0.70, indicating acceptable test-retest reliability. The ICC estimates for the remaining domain scores did not achieve the prespecified success criteria.

**Table 2:** QLQ-OES18 test-retest reliability

QLQ-OES18 Domain	*N*	ICC(A,1)
Dysphagia	139	**0.77**
Eating	138	**0.78**
Reflux	139	0.64
Pain	139	**0.76**
Dry mouth	139	0.48
Trouble with coughing	139	0.47
Swallowing saliva	139	0.53
Choke when swallowing	139	0.41
Trouble with taste	138	0.55
Trouble with talking	139	0.64
Index scale	138	**0.78**

Note. Reliability estimates are based on baseline versus week 3 follow-up. Bold text indicates those estimates that reached the acceptable threshold (≥ 0.70)

Abbreviations. ICC: intraclass correlation coefficient; QLQ-OES18: Quality of Life Questionnaire – Oesophageal Cancer 18-question module

### Construct validity

The concurrent validity estimates for the QLQ-OES18 are presented in Table 3. Convergent validators based on the QLQ-C30 were expected to correlate with QLQ-OES18 scores with an absolute value of ≥ 0.40, whereas discriminant validators were not expected to correlate with the QLQ-OES18. Except for the QLQ-C30 index score, there were no validator correlations with the QLQ-OES18 dysphagia, dry mouth, swallowing saliva, choke when swallowing, or trouble with talking domains that met the prespecified criterion. Other than the QLQ-C30 index score, only one of the validators met the criterion for each of the QLQ-OES18 trouble with coughing, trouble with taste, and reflux domains (as hypothesized, the QLQ-C30 dyspnea item met the criterion with trouble with coughing and the QLQ-C30 appetite loss item with trouble with taste). Most validators met the criterion for the remaining QLQ-OES18 eating and pain domains (including eating with QLQ-C30 appetite loss and pain with QLQ-C30 pain, as hypothesized), as well as the index score (9, 10, and 13 of the 16 validators, respectively).

**Table 3:** QLQ-OES18 concurrent validity at baseline

QLQ-C30 Validator	QLQ-OES18 Domain										
	Dry Mouth	Eating	Trouble With Coughing	Dysphagia	Pain	Reflux	Swallowing Saliva	Choke When Swallowing	Trouble With Taste	Trouble With Talking	Index Scale
Physical functioning	−0.32	−**0.46**	−0.36	−0.17	−**0.48**	−0.26	−0.30	−0.16	−0.36	−0.23	−**0.52**
Role functioning	−0.30	−**0.45**	−0.27	−0.13	−**0.43**	−0.29	−0.21	−0.13	−0.33	−0.22	−**0.46**
Emotional functioning	−0.29	−**0.45**	−0.32	−0.14	−**0.45**	−0.31	−0.25	−0.23	−0.32	−0.19	−**0.49**
Cognitive functioning	−0.34	−**0.42**	−0.34	−0.19	−**0.49**	−0.34	−0.32	−0.18	−0.39	−0.28	−**0.55**
Social functioning	−0.28	−0.36	−0.27	−0.10	−0.31	−0.29	−0.23	−0.15	−0.30	−0.20	−**0.42**
Fatigue	0.38	**0.51**	0.39	0.18	**0.55**	0.39	0.27	0.21	0.37	0.27	**0.59**
Nausea and vomiting	0.16	**0.49**	0.38	0.17	**0.46**	**0.46**	0.14	0.23	0.26	0.18	**0.49**
Pain	0.30	0.35	0.30	0.08	**0.59**	0.33	0.15	0.17	0.36	0.20	**0.46**
Dyspnea	0.29	0.32	**0.42**	0.12	0.33	0.26	0.23	0.14	0.21	0.28	**0.44**
Insomnia	0.19	0.30	0.27	0.08	0.35	0.32	0.18	0.13	0.24	0.17	0.37
Appetite loss	0.30	**0.55**	0.24	0.18	**0.42**	0.38	0.16	0.15	**0.48**	0.14	**0.51**
Constipation	0.29	0.31	0.30	0.10	0.34	0.34	0.18	0.11	0.34	0.23	**0.42**
Diarrhea	0.10	0.18	0.16	0.10	0.18	0.26	0.03	0.06	0.04	0.06	0.20
Financial difficulties	0.19	0.10	0.10	−0.05	0.10	0.21	0.08	0.19	0.14	0.12	0.19
GHS/QoL	−0.28	−**0.46**	−0.30	−0.20	−**0.45**	−0.28	−0.30	−0.18	−0.33	−0.20	−**0.51**
Index scale	**0.41**	**0.58**	**0.46**	0.17	**0.61**	**0.50**	0.29	0.25	**0.47**	0.31	**0.68**

Note. Bold text indicates those estimates that reached the prespecified threshold for acceptable correlations (|r| ≥ 0.40)

Abbreviations. GHS/QoL: global health status/quality of life; QLQ-C30: Quality of Life Questionnaire – Core 30; QLQ-OES18: Quality of Life Questionnaire – Oesophageal Cancer 18-question module

The known-groups validity estimates for the QLQ-OES18 are presented in Table 4. The mean difference in QLQ-OES18 scores were calculated between known groups defined by geographic region (Asia versus US/EU), baseline ECOG performance status (0 versus 1), and QLQ-C30 GHS/QoL items 29 and 30 (ratings of 1–6 versus 7). For geographic region, as expected, patients in the US/EU reported significantly higher mean QLQ-OES18 scores than patients in Asia for the domains of eating, pain, swallowing saliva, trouble with taste, and index score. These differences were associated with effect sizes indicating 20–61% explained variance. The remaining QLQ-OES18 domains demonstrated higher yet not significantly different mean scores for patients in the US/EU compared with patients in Asian, except for reflux and choke when swallowing, which demonstrated an unexpected reverse but nonsignificant trend (i.e., patients in Asia reported higher symptom scores than patients in the US/EU at baseline).

**Table 4:** QLQ-OES18 Known-Groups validity at baseline

QLQ-OES18 Domain	Known-Groups Validator	*N*	Group Mean Difference	SE	95% Confidence Interval	*P*-Value	Effect Size (ω^2^)
Dry mouth	**Region**		2.09	2.58	−2.99, 7.16	0.4196	0.394
	Asia	392					
	United States/Europe	97					
	**ECOG PS**		7.91	2.35	3.29, 12.53	0.0008	0.406
	0	124					
	1	378					
	**GHS/QoL item 29**		−11.82	3.63	−18.95, − 4.68	0.0012	0.405
	1–6	447					
	7	41					
	**GHS/QoL item 30**		−8.68	3.01	−14.6, − 2.76	0.0041	0.402
	1–6	425					
	7	63					
Eating	**Region**		11.30	2.31	6.77, 15.84	< 0.0001	0.477
	Asia	392					
	United States/Europe	97					
	**ECOG PS**		6.10	2.15	1.88, 10.33	0.0048	0.459
	0	124					
	1	378					
	**GHS/QoL item 29**		−12.77	3.30	−19.25, − 6.29	0.0001	0.466
	1–6	447					
	7	41					
	**GHS/QoL item 30**		−11.34	2.72	−16.69, − 6.00	< 0.0001	0.469
	1–6	425					
	7	63					
Trouble with coughing	**Region**		1.22	2.48	−3.65, 6.08	0.6236	0.238
	Asia	392					
	United States/Europe	97					
	**ECOG PS**		4.43	2.27	−0.03, 8.89	0.0515	0.243
	0	124					
	1	378					
	**GHS/QoL item 29**		−8.79	3.50	−15.65, − 1.93	0.0122	0.247
	1–6	447					
	7	41					
	**GHS/QoL item 30**		−8.45	2.89	−14.11, − 2.78	0.0036	0.251
	1–6	425					
	7	63					
Dysphagia	**Region**		0.58	3.99	−7.26, 8.42	0.8845	0.502
	Asia	392					
	United States/Europe	97					
	**ECOG PS**		−0.87	3.69	−8.12, 6.38	0.8137	0.501
	0	124					
	1	378					
	**GHS/QoL item 29**		0.17	5.69	−11.02, 11.36	0.9763	0.501
	1–6	447					
	7	41					
	**GHS/QoL item 30**		−6.05	4.70	−15.29, 3.19	0.1988	0.503
	1–6	425					
	7	63					
Pain	**Region**		5.60	1.88	1.92, 9.29	0.0030	0.349
	Asia	392					
	United States/Europe	97					
	**ECOG PS**		4.31	1.74	0.90, 7.73	0.0134	0.345
	0	124					
	1	378					
	**GHS/QoL item 29**		−11.15	2.65	−16.36, − 5.94	< 0.0001	0.360
	1–6	447					
	7	41					
	**GHS/QoL item 30**		−10.76	2.18	−15.04, − 6.48	< 0.0001	0.368
	1–6	425					
	7	63					
Reflux	**Region**		−0.26	2.03	−4.25, 3.72	0.8974	0.329
	Asia	392					
	United States/Europe	97					
	**ECOG PS**		5.64	1.86	1.99, 9.29	0.0025	0.341
	0	124					
	1	378					
	**GHS/QoL item 29**		−6.51	2.88	−12.16, − 0.85	0.0242	0.336
	1–6	447					
	7	41					
	**GHS/QoL item 30**		−6.11	2.38	−10.78, − 1.44	0.0104	0.338
	1–6	425					
	7	63					
Swallowing saliva	**Region**		7.16	2.69	1.87, 12.45	0.0081	0.197
	Asia	392					
	United States/Europe	97					
	**ECOG PS**		8.34	2.48	3.47, 13.21	0.0008	0.204
	0	124					
	1	378					
	**GHS/QoL item 29**		−2.69	3.87	−10.29, 4.91	0.4870	0.186
	1–6	447					
	7	41					
	**GHS/QoL item 30**		−8.24	3.18	−14.48, − 1.99	0.0098	0.196
	1–6	425					
	7	63					
Choke when swallowing	**Region**		−2.43	2.35	−7.05, 2.19	0.3021	0.322
	Asia	392					
	United States/Europe	97					
	**ECOG PS**		4.21	2.17	−0.05, 8.47	0.0528	0.326
	0	124					
	1	378					
	**GHS/QoL item 29**		−8.41	3.34	−14.97, − 1.85	0.0121	0.329
	1–6	447					
	7	41					
	**GHS/QoL item 30**		−4.77	2.77	−10.21, 0.67	0.0857	0.324
	1–6	425					
	7	63					
Trouble with taste	**Region**		7.60	2.51	2.67, 12.52	0.0026	0.214
	Asia	392					
	United States/Europe	97					
	**ECOG PS**		4.14	2.31	−0.41, 8.68	0.0743	0.204
	0	124					
	1	378					
	**GHS/QoL item 29**		−10.28	3.55	−17.26, − 3.31	0.0039	0.213
	1–6	447					
	7	41					
	**GHS/QoL item 30**		−10.86	2.92	−16.6, − 5.12	0.0002	0.221
	1–6	425					
	7	63					
Trouble with talking	**Region**		0.94	2.43	−3.83, 5.71	0.6992	0.193
	Asia	392					
	United States/Europe	97					
	**ECOG PS**		7.45	2.22	3.09, 11.81	0.0008	0.210
	0	124					
	1	378					
	**GHS/QoL item 29**		−6.97	3.45	−13.75, − 0.20	0.0438	0.199
	1–6	447					
	7	41					
	**GHS/QoL item 30**		−2.89	2.86	−8.51, 2.74	0.3137	0.194
	1–6	425					
	7	63					
Index scale	**Region**		3.26	1.45	0.42, 6.10	0.0246	0.608
	Asia	392					
	United States/Europe	97					
	**ECOG PS**		5.13	1.31	2.56, 7.70	0.0001	0.615
	0	124					
	1	378					
	**GHS/QoL item 29**		−7.89	2.02	−11.86, − 3.92	0.0001	0.615
	1–6	447					
	7	41					
	**GHS/QoL item 30**		−7.78	1.66	−11.04, − 4.52	< 0.0001	0.62
	1–6	425					
	7	63					

Note. The geographic region validator compared patients in Asia with patients in the US/EU. The ECOG PS validator compared 0 to 1. The QLQ-C30 GHS/QoL item 29 and item 30 validators compared ratings of 1 to 6 to 7

Abbreviations. SE: standard error; ECOG PS: Eastern Cooperative Oncology Group performance status; GHS/QoL: global health status/quality of life; QLQ-C30: Quality of Life Questionnaire – Core 30; QLQ-OES18: Quality of Life Questionnaire – Oesophageal Cancer 18-question module

As expected, patients with a baseline ECOG performance status of 1 had significantly higher mean QLQ-OES18 scores than patients with an ECOG performance status of 0 for the domains of dry mouth, eating, pain, reflux, swallowing saliva, trouble with talking, and index. These differences were associated with effect sizes indicating 20–62% explained variance. A nonsignificant trend was observed for the remaining domains, except for dysphagia.

As hypothesized, patients with a rating of “excellent” on QLQ-C30 GHS/QoL item 29 at baseline had significantly lower mean QLQ-OES18 scores (i.e., fewer symptoms) than those with a rating of “very poor” or any of the other ratings (i.e., 2–6) for most QLQ-OES18 domains (dry mouth, eating, trouble with coughing, pain, reflux, choke when swallowing, trouble with taste, trouble with talking, and index). These differences were associated with effect sizes indicating 20–62% explained variance. The swallowing saliva domain demonstrated the expected direction of effect, although the mean difference was nonsignificant. The dysphagia domain demonstrated no difference in mean scores between the QLQ-C30 GHS/QoL item 29 groups.

Results for known groups defined on QLQ-C30 GHS/QoL item 30 were consistent with those presented above for the QLQ-C30 GHS/QoL item 29.

### Responsiveness

#### Ability to detect change

Results from the primary analysis showed clear differentiation of QLQ-OES18 change scores between improvement and maintenance groups for the domains of dysphagia, pain, trouble with coughing, trouble with talking, and the index score (complete estimates are presented in Table 5). As expected, each of the effect size estimates was quite small (< 5%) due to large variability in these data, as indicated by the 95% CIs. Nonsignificant changes were observed between improvement and maintenance groups for the domains of eating, reflux, dry mouth, swallowing saliva, choke when swallowing, and trouble with taste; the estimated changes for these domains were in the expected direction, except for the domains of dry mouth, swallowing saliva, and choke when swallowing. No significant differentiation was observed between deterioration and maintenance groups; however, the direction of change was as expected for 50% of domains (excluding the index score), including eating, pain, dry mouth, trouble with taste, and trouble with talking.

**Table 5:** QLQ-OES18 change scores from baseline to week 9 by QLQ-C30 GHS/QoL anchor group: primary analysis

QLQ-OES18 Domain	Contrast (Anchor)	Group Mean Difference	SE	95% Confidence Interval	*P*-Value		Effect Size (Omnibus ω)
Dry mouth	Deterioration (*n* = 34) vs. maintenance (*n* = 79)	1.19	3.86	−6.37, 8.75	0.7560		
	Improvement (*n* = 26) vs. maintenance (*n* = 79)	1.33	4.27	−7.03, 9.7	0.7526		
							0.215
Eating	Deterioration (*n* = 34) vs. maintenance (*n* = 79)	0.31	2.78	−5.14, 5.77	0.9092		
	Improvement (*n* = 26) vs. maintenance (*n* = 79)	−5.31	3.08	−11.34, 0.73	0.0845		
							0.13
Trouble with coughing	Deterioration (*n* = 34) vs. maintenance (*n* = 79)	−4.82	3.39	−11.47, 1.84	0.1545		
	Improvement (*n* = 26) vs. maintenance (*n* = 79)	−8.72	3.75	−16.07, −1.38	0.0203		
							0.187
Dysphagia	Deterioration (*n* = 34) vs. maintenance (*n* = 79)	−3.79	4.69	−12.98, 5.39	0.4156		
	Improvement (*n* = 26) vs. maintenance (*n* = 79)	−11.3	5.21	−21.51, −1.08	0.0304		
							0.097
Pain	Deterioration (*n* = 34) vs. maintenance (*n* = 79)	1.16	1.98	−2.73, 5.05	0.5564		
	Improvement (*n* = 26) vs. maintenance (*n* = 79)	−6.2	2.19	−10.5, −1.89	0.0051		
							0.172
Reflux	Deterioration (*n* = 34) vs. maintenance (*n* = 79)	−0.75	2.37	−5.4, 3.9	0.7500		
	Improvement (*n* = 26) vs. maintenance (*n* = 79)	−2.81	2.62	−7.95, 2.34	0.2824		
							0.162
Swallowing saliva	Deterioration (*n* = 34) vs. maintenance (*n* = 79)	−1.45	3.97	−9.24, 6.34	0.7141		
	Improvement (*n* = 26) vs. maintenance (*n* = 79)	2.06	4.39	−6.54, 10.66	0.6360		
							0.385
Choke when swallowing	Deterioration (*n* = 34) vs. maintenance (*n* = 79)	−3.51	3.96	−11.27, 4.26	0.3736		
	Improvement (*n* = 26) vs. maintenance (*n* = 79)	2.58	4.38	−6.01, 11.17	0.5535		
							0.272
Trouble with taste	Deterioration (*n* = 34) vs. maintenance (*n* = 79)	4.58	2.97	−1.25, 10.41	0.1229		
	Improvement (*n* = 26) vs. maintenance (*n* = 79)	−5.82	3.30	−12.29, 0.65	0.0776		
							0.273
Trouble with talking	Deterioration (*n* = 34) vs. maintenance (*n* = 79)	3.19	2.45	−1.61, 7.98	0.1913		
	Improvement (*n* = 26) vs. maintenance (*n* = 79)	−6.09	2.72	−11.42, −0.76	0.0255		
							0.152
Index scale	Deterioration (*n* = 34) vs. maintenance (*n* = 79)	−0.31	1.54	−3.33, 2.72	0.8419		
	Improvement (*n* = 26) vs. maintenance (*n* = 79)	−4.2	1.71	−7.56, −0.85	0.0144		
							0.132

Note. Improvement was defined as a 1-point change in the QLQ-C30 GHS/QoL scale score, maintenance was defined as a 0-point change in the QLQ-C30 GHS/QoL scale score, and deterioration was defined as a 1-point change in the QLQ-C30 GHS/QoL scale score

Abbreviations: SE: standard error; GHS/QoL: global health status/quality of life; QLQ-C30: Quality of Life Questionnaire – Core 30; QLQ-OES18: Quality of Life Questionnaire – Oesophageal Cancer 18-question module

Results from the first sensitivity analysis that utilized broader definitions of improvement (≥ 1-point score increase) and deterioration (≥ 1-point score decrease) had a pattern similar to the results from the primary analysis, with the direction of change as expected for 8 of 11 domains (73%) (see Supplemental Table 4 in the Electronic supplementary material for the complete estimates from the sensitivity analysis). Significant differentiation between improvement and maintenance groups was observed for the QLQ-OES18 dysphagia, pain, trouble with coughing, trouble when talking, and index scores, which is consistent with findings from the primary analysis.

The results for the second sensitivity analysis are presented in Supplemental Table 5 in the Electronic supplementary material. The correlations between the QLQ-OES18 change scores and the anchor (QLQ-GHS/QoL) change scores ranged from − 0.09 to − 0.33. Only the correlation between the QLQ-OES18 index change score and the anchor change score met the prespecified criterion of ≥ 0.3 to demonstrate acceptable ability to detect change.

#### Estimation of meaningful change thresholds

Mean and median change from baseline in QLQ-OES18 scores according to QLQ-C30 GHS/QoL anchor change groups (i.e., deterioration, maintenance, and improvement) are presented in Table 6. The estimated deterioration thresholds of the QLQ-OES18 domains ranged from − 2.94 to 9.80-point change based on the 1-category deterioration anchor group, and − 2.83 to 19.44-point change based on the ≥ 2-category deterioration anchor group. As observed for both deterioration anchor groups, some QLQ-OES18 domains have a median change from baseline of zero and/or an unexpected negative point estimate. The authors posit the limitations associated with the selected anchor and that the trial was not powered to detect change in PRO endpoints, discussed below, have resulted in uninterpretable deterioration thresholds for several OES18 domain scores.

**Table 6:** QLQ-C30 GHS/QoL anchor-based within-patient meaningful deterioration and improvement thresholds for QLQ-OES18 change scores from baseline to week 9

QLQ-OES18 Domain	Anchor Group	Total Sample	Mean Threshold	Standard Deviation	Median Threshold
Dry mouth	2-point deterioration	14	14.29	28.39	16.67
	1-point deterioration	34	9.80	25.33	0.00
	Maintenance	79	1.27	20.29	0.00
	1-point improvement	26	−1.28	34.62	0.00
	2-point improvement	9	3.70	26.06	0.00
Eating	2-point deterioration	14	13.69	26.88	8.33
	1-point deterioration	34	5.64	13.72	0.00
	Maintenance	79	0.84	15.48	0.00
	1-point improvement	26	−3.53	20.84	0.00
	2-point improvement	9	−9.26	27.46	0.00
Trouble with coughing	2-point deterioration	14	4.76	17.82	0.00
	1-point deterioration	34	0.98	22.45	0.00
	Maintenance	79	6.33	17.76	0.00
	1-point improvement	26	−3.85	19.61	0.00
	2-point improvement	9	−7.41	14.70	0.00
Dysphagia	2-point deterioration	14	19.44	41.96	11.11
	1-point deterioration	34	−0.98	26.29	0.00
	Maintenance	79	4.92	29.60	0.00
	1-point improvement	26	−10.26	33.84	0.00
	2-point improvement	9	−12.35	35.77	0.00
Pain	2-point deterioration	14	0.79	15.39	0.00
	1-point deterioration	34	1.63	9.91	0.00
	Maintenance	79	1.55	11.90	0.00
	1-point improvement	26	−5.56	11.00	0.00
	2-point improvement	9	−16.05	26.71	−11.11
Reflux	2-point deterioration	14	5.95	15.48	0.00
	1-point deterioration	34	−2.45	13.69	0.00
	Maintenance	79	−0.21	15.90	0.00
	1-point improvement	26	−2.56	14.68	0.00
	2-point improvement	9	−9.26	22.22	0.00
Swallowing saliva	2-point deterioration	14	2.38	8.91	0.00
	1-point deterioration	34	0.00	21.71	0.00
	Maintenance	79	1.27	26.92	0.00
	1-point improvement	26	1.28	27.46	0.00
	2-point improvement	9	−22.22	44.10	0.00
Choke when swallowing	2-point deterioration	14	−2.38	15.82	0.00
	1-point deterioration	34	−2.94	15.06	0.00
	Maintenance	79	−0.84	24.45	0.00
	1-point improvement	26	−2.56	29.70	0.00
	2-point improvement	9	0.00	23.57	0.00
Trouble with taste	2-point deterioration	14	14.29	21.54	0.00
	1-point deterioration	34	7.84	27.29	0.00
	Maintenance	79	2.95	17.04	0.00
	1-point improvement	26	−5.13	18.12	0.00
	2-point improvement	9	−3.70	20.03	0.00
Trouble with talking	2-point deterioration	14	2.38	15.82	0.00
	1-point deterioration	34	3.92	13.64	0.00
	Maintenance	79	1.27	16.40	0.00
	1-point improvement	26	−7.69	19.57	0.00
	2-point improvement	9	−11.11	23.57	0.00
Index scale	2-point deterioration	14	7.56	11.22	6.53
	1-point deterioration	34	2.34	7.09	1.53
	Maintenance	79	1.93	8.50	0.83
	1-point improvement	26	−4.11	12.84	−1.67
	2-point improvement	9	−8.77	15.27	−2.22

Abbreviations. GHS/QoL: global health status/quality of life; QLQ-C30: Quality of Life Questionnaire – Core 30; QLQ-OES18: Quality of Life Questionnaire – Oesophageal Cancer 18-question module

None of the estimated QLQ-OES18 deterioration or improvement thresholds met the ± 33.33 criterion for characterizing change on a transformed score [30]. The largest deterioration thresholds were observed for the domains of dry mouth, dysphagia, and trouble with taste: 14.29 (median: 16.67), 19.44 (median: 11.11), and 14.29 (median: 0), respectively. The largest improvement thresholds were observed for the domains of pain and swallowing saliva, which were − 16.05 (median: −11.11) and − 22.22 (median: 0), respectively. Given that ± 33.33 is rather stringent, it is not surprising that this criterion was not achieved in this analysis within a population of patients with advanced or metastatic ESCC that present with low symptom severity at baseline (as measured by the QLQ-OES18). Further commentary on this observed finding is provided below in the Discussion and Limitation sections.

**Fig. 2:**
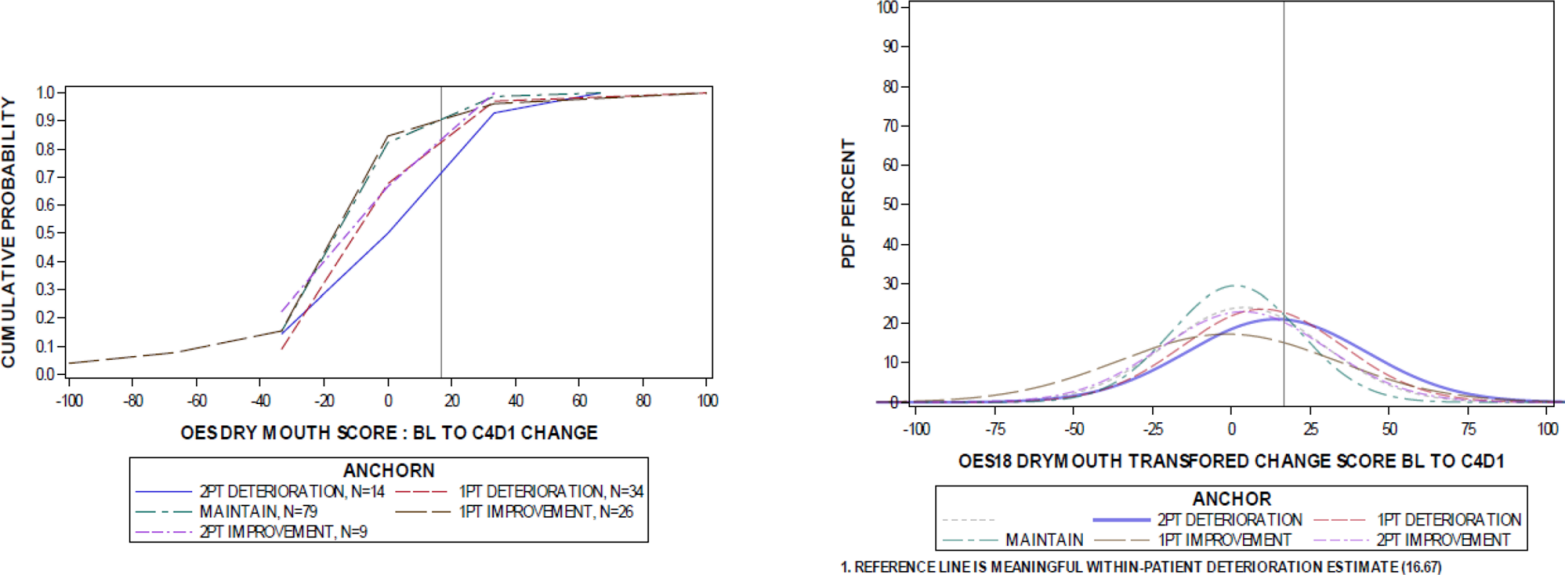
eCDF and ePDF of QLQ-OES18 dry mouth change scores from baseline to week 9 by anchor group. Abbreviations. BL: baseline; C4D1: cycle 4 day 1; eCDF: empirical cumulative distribution function; ePDF: empirical probability density function; PT: point; QLQ-OES18: Quality of Life Questionnaire – Oesophageal Cancer 18-question module.

The MWPC thresholds for QLQ-OES18 scores from Table 6 were visually explored with eCDF and ePDF figures. Separation between eCDF curves for each anchor group at the location of the MWPC threshold may suggest that the threshold is appropriate. eCDF and ePDF figures for the QLQ-OES18 dysphagia and dry mouth scores are presented as these domains came closest to meeting the FDA criterion for interpretability of thresholds (See Figs. 1 and 2). The eCDFs for dysphagia and dry mouth demonstrated graphical separation between the ≥ 2-category deterioration and maintenance anchor groups at the estimated thresholds. Although separation was observed, there is evidence of substantial overlap in the anchor group curves at the location of the point estimates as displayed in both the eCDF and ePDF figures for the dysphagia and dry mouth domains. Furthermore, the ePDF figures demonstrate that not all distributions are offset as expected, whereby deterioration (1-category change) is slightly skewed to the right (see Fig. 1) and improvement (≥ 2-category change) is slightly skewed to the left (see Fig. 2). These observations further suggest the anchor measure is suboptimal and that seeking to detect change in trials not designed to detect change in PROs yields uninterpretable evidence. Therefore, the interpretability of these estimated thresholds is limited.

The distribution-based estimates are presented in Supplemental Table 6 in the Electronic supplementary material. The estimates calculated for the 0.5 SD ranged between 6.27 and 17.43. The estimates calculated for the SEM ranged between 8.19 and 21.87. These ranges are consistent with those observed for anchor-based estimates, and reflect the point raised about design implications for findings in oncology trials not designed to detect PRO-based efficacy.

## Discussion

The present study examined the psychometric properties, namely reliability, construct validity, and responsiveness, of the EORTC QLQ-OES18 in the RATIONALE 302 trial population of patients with advanced or metastatic ESCC. Floor effects were demonstrated for all QLQ-OES18 items. While the presence of floor effects can affect measurement precision, the abundance of floor effects found in this study likely reflected the natural disease status at baseline and subsequent nature of ESCC progression over time. Reliability of some, not all, QLQ-OES18 domain scores met or exceeded the prespecified success criteria, both in terms of internal consistency and test-retest reliability. The reflux domain did not demonstrate adequate reliability; however, the estimate for internal consistency was within rounding distance of the success criterion. These findings are consistent with those of previous QLQ-OES18 validation studies, which reported lower alpha coefficients for reflux compared with the other OES18 scores [32, 33].

The correlations of the QLQ-OES18 scores with the convergent/discriminant predictors provided somewhat compelling evidence of the validity of the PROM, whereby the expected associations were generally observed. This provided evidence that the QLQ-OES18 measures constructs hypothesized to be similar or the same as the selected clinical validators. Similarly, the evaluation of the known-groups validity demonstrated that almost 90% of analyses showed the expected direction of effect. Evidence for the geographic region group was largely consistent with the hypothesized direction of effect. For domains that demonstrated an unexpected reverse effect (i.e., reflux and choke when swallowing), it is posited that the observed differences in scores between US/EU versus Asia could stem from dietary differences and/or variability in clinical management practices [34, 35].

Collectively, the examination of the measurement properties indicated that the EORTC QLQ-OES18 was able to reliably and validly measure patient-reported symptom severity in the RATIONALE 302 population. Ability to detect change and meaningful within-patient change evidence is reviewed next. However, given several limitations of the selected anchor and that RATIONALE 302 was not designed to detect efficacy in PRO-based endpoints, discussed in detail below in the Limitations section, these analyses should be considered exploratory and the results interpreted with caution.

For ability to detect change, our primary analyses indicated that some QLQ-OES18 domains were able to detect changes in symptom severity in this clinical trial population. Preliminary evidence of acceptable ability to detect improvement was observed for the QLQ-OES18 domains of dysphagia, pain, trouble with coughing, and trouble with talking, as well as the symptom index. Findings from the marginal mean contrasts demonstrated that none of the QLQ-OES18 domains met the predefined criteria for acceptable sensitivity in detecting deterioration; however, properly ordered effects were observed for the domains of eating, pain, dry mouth, trouble with taste, and trouble with talking. In addition, the second sensitivity analyses showed a low to no association between the QLQ-OES18 change scores and the anchor (GHS/QoL) change scores. Taken together, these findings appear to demonstrate mixed to limited support for the sensitivity of the QLQ-OES18 to robustly detect changes in symptoms within this patient population.

Thresholds of MWPC to support the interpretation of within-treatment arm score change were generated for all QLQ-OES18 domains and index. These anchor-based and distribution-based thresholds were found to be variable compared with the commonly used published threshold of ±10-points [11]. This suggests that the application of this historical threshold across EORTC scales and different types of cancer is suboptimal when characterizing within-subject clinically meaningful changes.

### Limitations

Patients in this trial reported only minimal symptom severity at baseline, which resulted in a restriction of the range across several QLQ-OES18 domain and item scores, as well as in the potential magnitude of improvement experienced by patients by week 9. In addition, there were no global health or symptom-specific anchors collected in this trial and no additional clinical outcomes/measures were fit for use. As such, the QLQ-C30 GHS/QoL score was selected as an anchor for responsiveness analyses. Although the QLQ-C30 GHS/QoL items demonstrated adequate correlation (≥ 0.30) with more than half of the QLQ-OES18 scores at baseline, these associations were not reflected when correlating the change scores. The global health concepts assessed in the QLQ-C30 GHS/QoL are not appropriately proximal to the concepts being measured by the QLQ-OES18 (e.g., specific symptoms) [36].

The responsiveness analyses and conclusions drawn from them are limited by the appropriateness of the QLQ-C30 GHS/QoL as an anchor variable. Per current FDA guidance, the responsiveness evaluation should “*examine the relationship between changes in the clinical outcome assessment’s scores and changes in some other measure(s) of the same or proximal construct*” [13]. While alternative approaches to utilizing the QLQ-C30 GHS/QoL as an anchor to identify meaningful change have been described [37], this scale does not satisfy current FDA guidance on appropriate anchor measures. Specifically, it is recommended that concept-specific Patient Global Impression of Change/Patient Global Impression of Severity or the corresponding Clinician Global Impression of Change/Clinician Global Impression of Severity measures are included in future studies for defining anchor groups.

The above limitation is also extended to the test-retest evaluation. The stable subgroup was predefined based on a change score of zero on the QLQ-C30 GHS/QoL at week 3. This criterion is far from ideal and is unlikely to appropriately capture patients who have stable disease during this retest interval. In addition, the estimates of test-retest reliability were calculated during an interval within the treatment period. As such, the estimates of test-retest reliability presented here should be interpreted with caution.

For the estimated MWPC thresholds, it is acknowledged that not all point estimates (mean or median) reflect observable values or plausible score increments on the underlying measurement scale (i.e., 6.67, 11.1, 22.2, …). It is important to highlight this observation, as it limits the utility of these thresholds to define ‘responders’ for the purposes of responder or time-to-event analyses. Recent work by Cocks & Buchanan provides a detailed appraisal, using EORTC QLQ-C30 as an example, of this issue [31]. As such, the OES18 MWPC thresholds generated in this analysis were not considered for use beyond the exploratory interpretation of longitudinal mean/median score changes within treatment arm.

In addition, we highlight that none of the estimated MWPC thresholds for the QLQ-C30 OES18 met ± 33.33-point change. It is acknowledged that this is a stringent criterion based on FDA guidance and, as such, this analysis aimed to test the feasibility of such a criterion [30]. Given this population consisted of patients with advanced or metastatic ESCC that present with low symptom severity at baseline (as measured by the QLQ-OES18), a large magnitude of change in symptom scores was not expected by week 9 (cycle 4, day 1).

Related to the low symptom severity at baseline, the lack of ability to detect change, and the lack of interpretable MWPC threshold estimates, and lack of separation in eCDF and ePDF curves, is the matter of trial design. In most oncology trials, primary and key secondary endpoints are defined on time to event outcomes (e.g., overall survival, progression-free survival, etc.) or overall response rate, etc. These trials are, of course, powered to detect efficacy for these endpoints and largely succeed in detecting powered efficacy. These trials are rarely powered to detect efficacy in PRO-based endpoints, particularly change from baseline mixed models. And where a study is not powered to detect effects, such effects are not detected, and if they are they should not be interpreted without extreme caution. For example, were such a trial to be designed to detect such PRO-based effects, one would employ inclusion criteria to maximize the symptom severity at baseline in order to detect efficacy for powered PRO-improvement endpoints. This is simply a reality that must be considered when endeavoring to interpret evidence of responsiveness in such trials. And yet, it is clear from the other analyses that the QLQ-OES18 is relevant to this population and psychometrically capable of reflecting the experience of patients with ESCC.

Finally, there were prespecified hypotheses regarding concurrent validity for some, but not all, of the QLQ-OES18 scores.

## Conclusions

Overall, a collection of psychometric evidence indicated that the EORTC QLQ-OES18 was able to reliably and validly measure patient-reported symptom severity in the RATIONALE 302 population. Additional work is needed to appropriately estimate anchor-based MWPC thresholds for the QLQ-OES18 within ESCC populations and this exercise would benefit from trials designed to detect such effects. Generation of robust MWPC thresholds will enable an accurate evaluation of within-treatment arm meaningful changes in key OES18-based endpoints in trials assessing the efficacy of novel treatments in patients with advanced or metastatic ESCC.

## Electronic supplementary material

Below is the link to the electronic supplementary material.

Supplementary Material 1

## Data Availability

BeOne Medicines voluntarily shares anonymous data on completed studies responsibly and provides qualified scientific and medical researchers access to anonymous data and supporting clinical trial documentation for clinical trials in dossiers for medicines and indications after submission and approval in the United States, China, and Europe. Clinical trials supporting subsequent local approvals, new indications, or combination products are eligible for sharing once corresponding regulatory approvals are achieved. BeOne Medicines shares data only when permitted by applicable data privacy and security laws and regulations. In addition, data can only be shared when it is feasible to do so without compromising the privacy of study participants. Qualified researchers may submit data requests/research proposals for BeOne Medicines review and consideration through BeOne Medicines’ Clinical Trial Webpage at https://www.beigene.com/our-science-and-medicines/our-clinical-trials/.

## Abbreviations

CI: Confidence Interval
ECOG: Eastern Cooperative Oncology Group
eCDF: Empirical Cumulative Distribution Function
EORTC: European Organisation for Research and Treatment of Cancer
ePDF: Empirical Probability Density Function
ESCC: Esophageal Squamous Cell Carcinoma
FDA: Food and Drug Administration
GHS/QoL: Global Health Status/Quality of Life
HRQoL: Health-Related Quality of Life
ICCs: Intraclass Correlation Coefficients
MWPC: Meaningful Within-Patient Change
PROM: Patient-Reported Outcome Measure
QLQ-C30: Quality of Life Questionnaire – Core 30
QLQ-OES18: Quality of Life Questionnaire – Oesophageal Cancer 18-question module

## Footnotes

1 For example, given a 5-item domain, consider the following response patterns to the 5 items at T1 and T2: 1,2,3,4,1 & 4,2,3,4,3. These responses correspond to EORTC raw scores of 2.2 and 3.2, respectively, and their difference is exactly 1 point: 3.2–2.2 = 1. Converting these raw scores to transformed scores using the EORTC algorithm yields transformed scores of 40 and 73.33, the difference being: 73.33–40.00 = 33.33.

2 An alternative means of defining the minimum change on a transformed score was considered by Cocks & Buchanan (2023) [31]. Those authors considered what the minimum transformed score change would be if only a single item contributing to a score changed one category and none of the other items changed at all. For example, given a 5-item domain, consider the following response patterns to the 5 items at T1 and T2: 1,2,3,4,1 & 1,2,3,4,2. Here, only the 5th item response changes from 1 to 2 between T1 and T2. These responses correspond to EORTC raw scores of 2.2 and 2.4, respectively, and their difference is exactly 0.2 points: 2.4–2.2 = 0.2. Converting these raw scores to transformed scores using the EORTC algorithm yields transformed scores of 40 and 46.67, the difference being: 46.67–40.00 = 6.67, which is the minimum change threshold presented by Cocks & Buchanan (2023) [31] in their Fig. 1. While related, the approach outlined in draft PFDD 3 (2018) [30] and taken in this analysis is distinct from that considered by Cocks & Buchanan (2023) [31].
