# Supplementary material for: Psychometric validation of the EORTC QLQ-OES18 in patients with advanced or metastatic esophageal squamous cell carcinoma
Source: J Patient Rep Outcomes. 2025 May 21;9:56. doi: 10.1186/s41687-025-00891-4 (PMC12095746; doi:10.1186/s41687-025-00891-4)
Supplement: Supplementary file 1 — Supplementary Material 1 [file 41687_2025_891_MOESM1_ESM.docx]

**Supplemental Table 1. QLQ-OES18 Item Response Distributions at Baseline**

| **QLQ-OES18 Item** | **Not at All**  **n (%)** | **A Little**  **n (%)** | **Quite a Bit**  **n (%)** | **Very Much**  **n (%)** |
| --- | --- | --- | --- | --- |
| Item 1: Could you eat solid food? | 118 (24.13) | 113 (23.11) | 86 (17.59) | 172 (35.17) |
| Item 2: Could you eat liquidized or soft food? | 84 (17.21) | 69 (14.14) | 72 (14.75) | 263 (53.89) |
| Item 3: Could you drink liquids? | 78 (15.95) | 57 (11.66) | 68 (13.91) | 286 (58.49) |
| Item 4: Have you had trouble with swallowing your saliva? | 374 (76.48) | 77 (15.75) | 23 (4.7) | 15 (3.07) |
| Item 5: Have you choked when swallowing? | 310 (63.39) | 152 (31.08) | 24 (4.91) | 3 (0.61) |
| Item 6: Have you had trouble enjoying your meals? | 267 (54.83) | 147 (30.18) | 49 (10.06) | 24 (4.93) |
| Item 7: Have you felt full up too quickly? | 261 (53.7) | 164 (33.74) | 40 (8.23) | 21 (4.32) |
| Item 8: Have you had trouble with eating? | 278 (57.08) | 141 (28.95) | 41 (8.42) | 27 (5.54) |
| Item 9: Have you had trouble with eating in front of other people? | 362 (74.79) | 92 (19.01) | 22 (4.55) | 8 (1.65) |
| Item 10: Have you had a dry mouth? | 265 (54.3) | 187 (38.32) | 29 (5.94) | 7 (1.43) |
| Item 11: Did food and drink taste different from usual? | 364 (74.74) | 95 (19.51) | 16 (3.29) | 12 (2.46) |
| Item 12: Have you had trouble with coughing? | 350 (71.72) | 103 (21.11) | 30 (6.15) | 5 (1.02) |
| Item 13: Have you had trouble with talking? | 375 (76.69) | 82 (16.77) | 25 (5.11) | 7 (1.43) |
| Item 14: Have you had acid indigestion or heartburn? | 349 (71.37) | 120 (24.54) | 16 (3.27) | 4 (0.82) |
| Item 15: Have you had trouble with acid or bile coming into your mouth? | 322 (65.85) | 139 (28.43) | 21 (4.29) | 7 (1.43) |
| Item 16: Have you had pain when you eat? | 364 (74.74) | 103 (21.15) | 15 (3.08) | 5 (1.03) |
| Item 17: Have you had pain in your chest? | 305 (62.37) | 151 (30.88) | 22 (4.5) | 11 (2.25) |
| Item 18: Have you had pain in your stomach? | 372 (76.07) | 95 (19.43) | 15 (3.07) | 7 (1.43) |

Notes. Items with floor effects are flagged in yellow (the response categories for items 1 to 3 are reverse scored, with “Not at All” reflecting the worst state and “Very Much” reflecting the best state; therefore, floor effects are reflected in the percentages of “Very Much” for these items). Items with a response category endorsement below 10% are flagged in orange.

Abbreviation. QLQ-OES18: Quality of Life Questionnaire – Oesophageal Cancer 18-question module.

**Supplemental Table 2. QLQ-OES18 Inter-Item Correlations at Baseline**

| **QLQ-OES18 Item** | **Item 1** | **Item 2** | **Item 3** | **Item 4** | **Item 5** | **Item 6** | **Item 7** | **Item 8** | **Item 9** | **Item 10** | **Item 11** | **Item 12** | **Item 13** | **Item 14** | **Item 15** | **Item 16** | **Item 17** | **Item 18** |
| --- | --- | --- | --- | --- | --- | --- | --- | --- | --- | --- | --- | --- | --- | --- | --- | --- | --- | --- |
| Item 1 | 1 | . | . | . | . | . | . | . | . | . | . | . | . | . | . | . | . | . |
| Item 2 | **0.82** | 1 | . | . | . | . | . | . | . | . | . | . | . | . | . | . | . | . |
| Item 3 | **0.72** | **0.93** | 1 | . | . | . | . | . | . | . | . | . | . | . | . | . | . | . |
| Item 4 | 0.36 | 0.24 | 0.24 | 1 | . | . | . | . | . | . | . | . | . | . | . | . | . | . |
| Item 5 | 0.25 | 0.08 | 0.09 | 0.37 | 1 | . | . | . | . | . | . | . | . | . | . | . | . | . |
| Item 6 | **0.47** | 0.28 | 0.26 | **0.51** | **0.56** | 1 | . | . | . | . | . | . | . | . | . | . | . | . |
| Item 7 | 0.13 | 0.10 | 0.13 | 0.17 | 0.23 | **0.48** | 1 | . | . | . | . | . | . | . | . | . | . | . |
| Item 8 | **0.49** | 0.35 | 0.36 | **0.58** | **0.57** | **0.82** | **0.43** | 1 | . | . | . | . | . | . | . | . | . | . |
| Item 9 | **0.40** | 0.36 | 0.31 | **0.48** | 0.38 | **0.68** | 0.28 | **0.72** | 1 | . | . | . | . | . | . | . | . | . |
| Item 10 | 0.14 | 0.12 | 0.12 | 0.38 | 0.22 | 0.28 | 0.39 | 0.35 | 0.30 | 1 | . | . | . | . | . | . | . | . |
| Item 11 | 0.28 | 0.21 | 0.16 | 0.34 | 0.24 | **0.54** | **0.52** | **0.47** | **0.49** | **0.51** | 1 | . | . | . | . | . | . | . |
| Item 12 | 0.17 | 0.10 | 0.07 | 0.39 | 0.30 | **0.47** | **0.44** | **0.40** | **0.44** | 0.29 | 0.4 | 1 | . | . | . | . | . | . |
| Item 13 | 0.19 | 0.19 | 0.12 | **0.52** | 0.28 | 0.38 | 0.21 | **0.42** | **0.46** | 0.26 | **0.41** | **0.54** | 1 | . | . | . | . | . |
| Item 14 | 0.19 | 0.13 | 0.17 | 0.22 | 0.25 | 0.31 | 0.35 | 0.33 | 0.29 | 0.29 | 0.39 | 0.39 | 0.20 | 1 | . | . | . | . |
| Item 15 | 0.13 | 0.14 | 0.08 | 0.25 | 0.33 | 0.37 | **0.40** | 0.33 | 0.32 | 0.22 | **0.40** | **0.41** | 0.38 | 0.65 | 1 | . | . | . |
| Item 16 | 0.28 | 0.27 | 0.26 | **0.49** | **0.48** | **0.61** | 0.34 | **0.67** | 0.49 | 0.33 | **0.53** | **0.42** | 0.38 | **0.44** | **0.43** | 1 | . | . |
| Item 17 | 0.23 | 0.24 | 0.23 | 0.36 | 0.30 | 0.39 | 0.38 | **0.47** | 0.36 | 0.26 | **0.44** | **0.46** | 0.27 | 0.39 | 0.33 | **0.59** | 1 | . |
| Item 18 | 0.07 | 0.12 | 0.10 | 0.18 | 0.17 | 0.36 | **0.52** | **0.42** | 0.24 | 0.31 | **0.46** | 0.30 | 0.09 | **0.47** | 0.39 | **0.65** | **0.50** | 1 |

Notes. The response categories for items 1 to 3 are reverse scored to maintain consistency with the rest of the symptom scores. Bold text indicates those estimates that reached the prespecified threshold for acceptable correlations (|r| ≥ 0.40).

Abbreviation. QLQ-OES18: Quality of Life Questionnaire – Oesophageal Cancer 18-question module.

**Supplemental Table 3. QLQ-OES18 Internal Consistency: Item Level**

| **QLQ-OES18 Item/Domain** | **Total Score Correlation** | **Item-Level Cronbach Alpha** |
| --- | --- | --- |
| Item 1: Dysphagia | 0.65 | **0.91** |
| Item 2: Dysphagia | **0.86** | **0.73** |
| Item 3: Dysphagia | **0.77** | **0.81** |
| Item 6: Eating | **0.72** | 0.63 |
| Item 7: Eating | 0.36 | **0.82** |
| Item 8: Eating | **0.71** | 0.64 |
| Item 9: Eating | 0.52 | **0.74** |
| Item 14: Reflux | 0.50 | . |
| Item 15: Reflux | 0.50 | . |
| Item 16: Pain | 0.59 | 0.56 |
| Item 17: Pain | 0.49 | 0.68 |
| Item 18: Pain | 0.53 | 0.63 |
| Item 1: Index scale | 0.17 | **0.81** |
| Item 2: Index scale | 0.04 | **0.80** |
| Item 3: Index scale | 0.02 | **0.80** |
| Item 4: Index scale | 0.34 | **0.78** |
| Item 5: Index scale | 0.37 | **0.77** |
| Item 6: Index scale | 0.57 | **0.76** |
| Item 7: Index scale | 0.47 | **0.77** |
| Item 8: Index scale | 0.59 | **0.76** |
| Item 9: Index scale | 0.43 | **0.77** |
| Item 10: Index scale | 0.37 | **0.77** |
| Item 11: Index scale | 0.52 | **0.76** |
| Item 12: Index scale | 0.47 | **0.77** |
| Item 13: Index scale | 0.34 | **0.78** |
| Item 14: Index scale | 0.42 | **0.77** |
| Item 15: Index scale | 0.47 | **0.77** |
| Item 16: Index scale | 0.57 | **0.76** |
| Item 17: Index scale | 0.47 | **0.77** |
| Item 18: Index scale | 0.44 | **0.77** |

Notes. Bold text indicates those estimates that reached the prespecified acceptable threshold (α ≥ 0.70). Item-level Cronbach alpha values are missing for the reflux domain because this domain only has 2 items, and the value is computed by the subsequent removal of each item. The dysphagia domain items were reverse scored to maintain consistency with the rest of the symptom scores. Item-total correlations were corrected for overlap.

Abbreviation. QLQ-OES18: Quality of Life Questionnaire – Oesophageal Cancer 18-question module.

**Supplemental Table 4.** **QLQ-OES18 Change Scores From Baseline to Week 9 by QLQ-C30 GHS/QoL Anchor Group (Sensitivity Analysis)**

| **QLQ-OES18 Domain** | **Contrast (Anchor)** | **Group Mean**  **Difference** | **95% Confidence Interval** | **P-Value** |
| --- | --- | --- | --- | --- |
| Dry mouth | Deterioration vs. maintenance | 3.42 | –3.52, 10.36 | 0.3319 |
|  | Improvement vs. maintenance | –0.76 | –8.05, 6.53 | 0.8373 |
| Eating | Deterioration vs. maintenance | 3.37 | –1.77, 8.50 | 0.1976 |
|  | Improvement vs. maintenance | –7.36 | –12.81, –1.92 | 0.0083 |
| Trouble with coughing | Deterioration vs. maintenance | –1.86 | –7.91, 4.19 | 0.5445 |
|  | Improvement vs. maintenance | –9.87 | –16.22, –3.53 | 0.0024 |
| Dysphagia | Deterioration vs. maintenance | 2.53 | –5.66, 10.72 | 0.5423 |
|  | Improvement vs. maintenance | –11.38 | –20.00, –2.77 | 0.0099 |
| Pain | Deterioration vs. maintenance | 1.92 | –1.56, 5.41 | 0.2773 |
|  | Improvement vs. maintenance | –6.69 | –10.36, –3.01 | 0.0004 |
| Reflux | Deterioration vs. maintenance | 1.76 | –2.39, 5.90 | 0.4043 |
|  | Improvement vs. maintenance | –3.79 | –8.15, 0.58 | 0.0886 |
| Swallowing saliva | Deterioration vs. maintenance | –1.9 | –8.27, 4.48 | 0.5577 |
|  | Improvement vs. maintenance | –2.39 | –9.08, 4.31 | 0.4828 |
| Choke when swallowing | Deterioration vs. maintenance | –2.56 | –8.9, 3.79 | 0.4275 |
|  | Improvement vs. maintenance | 0.62 | –6.05, 7.28 | 0.8556 |
| Trouble with taste | Deterioration vs. maintenance | 4.61 | –0.84, 10.05 | 0.0967 |
|  | Improvement vs. maintenance | –4.86 | –10.63, 0.91 | 0.0984 |
| Trouble with talking | Deterioration vs. maintenance | 2.69 | –1.72, 7.09 | 0.2303 |
|  | Improvement vs. maintenance | –6.84 | –11.48, –2.19 | 0.0041 |
| Index scale | Deterioration vs. maintenance | 1.56 | –1.1, 4.22 | 0.2495 |
|  | Improvement vs. maintenance | –5.78 | –8.6, –2.95 | <0.0001 |

Note. Improvement was defined as a ≥1-point change in the QLQ-C30 GHS/QoL scale score, maintenance was defined as a 0-point change in the QLQ-C30 GHS/QoL scale score, and deterioration was defined as a ≥1-point change in the QLQ-C30 GHS/QoL scale score.

Abbreviations. GHS/QoL: global health status/quality of life; QLQ-C30: Quality of Life Questionnaire – Core 30; QLQ-OES18: Quality of Life Questionnaire – Oesophageal Cancer 18-question module.

**Supplemental Table 5.** **Correlations Between QLQ-OES18 and QLQ-C30 GHS/QoL Change From Baseline to Week 9 Scores**

| **QLQ-OES18 Domain** | **QLQ-C30 GHS/QoL** |
| --- | --- |
| Dry mouth | –0.17 |
| Eating | –0.29 |
| Trouble with coughing | –0.14 |
| Dysphagia | –0.18 |
| Pain | –0.29 |
| Reflux | –0.13 |
| Swallowing saliva | –0.09 |
| Choke when swallowing | 0.02 |
| Trouble with taste | –0.20 |
| Trouble with talking | –0.20 |
| Index scale | –0.33 |

Abbreviations. GHS/QoL: global health status/quality of life; QLQ-C30: Quality of Life Questionnaire – Core 30; QLQ-OES18: Quality of Life Questionnaire – Oesophageal Cancer 18-question module.

**Supplemental Table 6. QLQ-OES18 Distribution-Based Estimates**

| **QLQ-OES18 Domain** | **0.5*SD** | **SEM^†^** |
| --- | --- | --- |
| Dry mouth | 11.23 | 19.15 |
| Eating | 10.24 | 14.17 |
| Trouble with coughing | 10.76 | 17.05 |
| Dysphagia | 17.43 | 20.46 |
| Pain | 8.27 | 12.71 |
| Reflux | 8.86 | 12.27 |
| Swallowing saliva | 11.84 | 21.87 |
| Choke when swallowing | 10.28 | 16.92 |
| Trouble with taste | 10.96 | 17.93 |
| Trouble with talking | 10.60 | 12.43 |
| Index scale | 6.27 | 8.19 |

**^†^**SEM defined as SD * sqrt(1 - reliability); ICC(A,1) used to define reliability

Abbreviations. SD: Standard Deviation; SEM: Standard Error of Measurement; QLQ-OES18: Quality of Life Questionnaire – Oesophageal Cancer 18-question module.
